# The impact of confounding on the associations of different adiposity measures with the incidence of cardiovascular disease: a cohort study of 296 535 adults of white European descent

**DOI:** 10.1093/eurheartj/ehy057

**Published:** 2018-03-16

**Authors:** Stamatina Iliodromiti, Carlos A Celis-Morales, Donald M Lyall, Jana Anderson, Stuart R Gray, Daniel F Mackay, Scott M Nelson, Paul Welsh, Jill P Pell, Jason M R Gill, Naveed Sattar

**Affiliations:** 1 Department of Obstetrics and Gynaecology, School of Medicine, University of Glasgow, Level 2, New Lister Building, 10-16 Alexandra Parade, Glasgow G31 2ER, UK; 2 Institute of Cardiovascular and Medical Sciences, University of Glasgow, University Avenue, Glasgow G12 8TA, UK; 3 Institute of Health and Wellbeing, University of Glasgow, 1 Lilybank Gardens, Glasgow G12 8RZ, UK

**Keywords:** Incidence, Cardiovascular disease, BMI, Percentage body fat mass, Adiposity, Bias

## Abstract

**Aims:**

The data regarding the associations of body mass index (BMI) with cardiovascular (CVD) risk, especially for those at the low categories of BMI, are conflicting. The aim of our study was to examine the associations of body composition (assessed by five different measures) with incident CVD outcomes in healthy individuals.

**Methods and results:**

A total of 296 535 participants (57.8% women) of white European descent without CVD at baseline from the UK biobank were included. Exposures were five different measures of adiposity. Fatal and non-fatal CVD events were the primary outcome. Low BMI (≤18.5 kg m^−2^) was associated with higher incidence of CVD and the lowest CVD risk was exhibited at BMI of 22–23 kg m^−2^ beyond, which the risk of CVD increased. This J-shaped association attenuated substantially in subgroup analyses, when we excluded participants with comorbidities. In contrast, the associations for the remaining adiposity measures were more linear; 1 SD increase in waist circumference was associated with a hazard ratio of 1.16 [95% confidence interval (CI) 1.13–1.19] for women and 1.10 (95% CI 1.08–1.13) for men with similar magnitude of associations for 1 SD increase in waist-to-hip ratio, waist-to-height ratio, and percentage body fat mass.

**Conclusion:**

Increasing adiposity has a detrimental association with CVD health in middle-aged men and women. The association of BMI with CVD appears more susceptible to confounding due to pre-existing comorbidities when compared with other adiposity measures. Any public misconception of a potential ‘protective’ effect of fat on CVD risk should be challenged.

**Take home figure:**
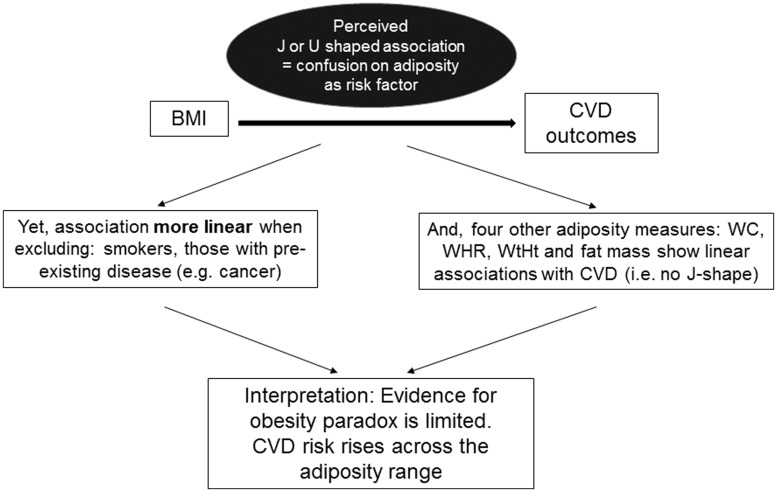
The obesity paradox is mainly due to the effect of confounding on BMI and disappears on other adiposity measures.

## Introduction

The obesity epidemic is an emerging public health problem with substantial consequences for health care expenditure and overall quality of life and wellbeing.^1^ Recent data from the World Health Organisation suggest that over half of the adult population worldwide is currently overweight [body mass index (BMI) ≥25 kg m^−2^] or obese (BMI ≥30 kg m^−2^),^2^ and researchers project that the obesity prevalence will increase by 33% over the next two decades impacting further on global health burden and cost.^3^ A large body of data have underscored the deleterious effect of high BMI on the risk of myocardial infarction, stroke, cancer, and overall mortality.^4–9^ However, there is a large body of evidence supporting the so-called ‘obesity paradox’ concept, which posits that being overweight or even obese is ‘protective’ of, or has no impact on, cardiovascular (CVD), and overall mortality,^10^ especially in elderly individuals or with diagnosed coronary disease or other severe medical conditions.^11^^,^^12^ The obesity paradox is also discussed in recent ESC CVD prevention guidelines^13^ in relation to people with existing CVD so more clarity on this topic is needed.

The confusion about the relationship of adiposity with adverse outcomes is exacerbated by data reporting that low BMI (<20 kg m^−2^) is associated with increased risk of CVD, cancer, and all-cause mortality relative to BMI between 20 and 25 kg m^−2^.^14^ These data, along with other data supporting the obesity paradox, could lead to the confusion that being overweight or obese may be protective against CVD compared with having a normal weight. The reasons for these paradoxical findings are unknown, but issues with the use of BMI to quantify weight status may contribute. Indeed, BMI may be more susceptible to the impact of disease,^15^ compared with other adiposity measures, as individuals with subclinical disease may lose weight due to muscle mass loss before a clinical diagnosis is made,^16^ whereas percentage body fat mass may continue to increase and fat distribution may not necessarily change. Furthermore, BMI has limited value in distinguishing between lean and fat mass or accounting for fat distribution,^17^ as central adiposity (i.e. waist circumference) rather than BMI correlates more strongly with visceral fat.^18^

The lack of robust data linking other body composition measures, on top of BMI, with long-term outcomes has further contributed to the persistence and re-stating of conflicting findings that can result in erroneous, or at least potentially confusing, public health messages. UK Biobank is a large, well phenotyped cohort with a variety of body composition measures, which allows us to study the above associations in well selected groups attenuating reverse causality bias. Therefore, the aim of our study was to examine the continuous associations of several body composition measures (BMI, waist circumference, waist-to-hip ratio, waist-to-height ratio, and percentage body fat) with the incidence of CVD (morbidity and mortality) in healthy individuals.

## Methods

### Study design and participants

UK Biobank is a large prospective study which, between 2006 and 2010, recruited 502 664 participants (response rate 5.5%), aged 40–69 years, who consented for their records to be linked with routine data (national hospital and death registries). Participants attended one of 22 assessment centres across the UK where completed a touch screen questionnaire, had physical measurement taken and provided biological samples as described in detail elsewhere.^19^^,^^20^ At the time of analysis, follow up was available for both hospital and death data up to August 15th 2015 for England, August 14th 2015 for Wales, and June 22nd 2015 for Scotland. Participants of non-White European descent were excluded from all the analyses (*n* = 28 892) as ethnicity modifies the association between adiposity and vascular health. Participants with any CVD at baseline [self-reported or from hospital records (ICD I00-I99)] were excluded from the analyses looking at the association of body composition markers with incidence of CVD (*n* = 170 058). A landmark analysis of events occurring from 2 years after recruitment was conducted to minimise the impact of reverse causality on the associations with the incidence of CVD.

### Exposures

Exposures were five adiposity markers; BMI is the ratio of the measured body mass in kg divided by the squared height measured in metres. Height was measured using a Seca 202 height measure. Weight was measured to the nearest 0.1 kg using the Tanita BC-418 MA body composition analyser. The natural indent was measured for the waist circumference (the umbilicus was used if the natural indent could not be observed). The hip circumference was recorded at the widest part of the hips. Waist-to-hip ratio and waist-to-height ratio are the ratios of waist-to-hip circumference and waist circumference to height, respectively. Percentage body fat was measured using the Tanita BC-418 MA body composition analyser (fat mass divided by the total body mass). All exposures were treated as continuous variables. Body mass index of 22 kg m^−2^ was the referent value for BMI.^6^ The referent value for the other adiposity measure was the corresponding value of each measure for BMI of 22 kg m^−2^ by regressing the BMI on each adiposity measure.

### Outcomes

Information about incident CVD after recruitment was available from hospital and death registries. These registries use the International Classification of Diseases system version 10 (ICD-10) for coding outcomes. The primary outcome was CVD (fatal and non-fatal events); defined as an ICD 10 code of I00-I99. Secondary outcomes were CVD mortality, non-fatal CVD events, and a composite outcome of ischaemic heart and cerebrovascular events (ICD I20-I25 and ICD I61-I67 and I69).

### Other variables

Age was treated as a continuous variable. Tobacco smoking (never smokers, current smokers, and ex-smokers), frequency of alcohol intake (daily, three to four times a week, once or twice a week, less frequently than once a month, or never), and diabetes diagnosed by a doctor (yes, no) were self-reported. Townsend quintiles (measure of deprivation based on four census variables; unemployment, non-car ownership, non-house ownership, and household overcrowding) and educational qualifications (higher degree, any school degree, vocational qualifications such as Higher National Certificate or Higher National Diploma, other qualifications, or none of the above) were used as measures of socioeconomic status. Moderate to vigorous physical activity (MVPA) was calculated in minutes per day (continuous) based on the answers in short version of the International Physical Activity Questionnaire and was truncated to 360 min per day (if reported higher). MVPA was used because of its direct associations with CVD.^21^ Systolic blood pressure was the average of two readings taken by trained personnel.

### Statistical analysis

We used multivariable cubic regression splines to model the associations between adiposity measures and each outcome using the mvrs command in Stata. When there was evidence of non-linearity, we firstly created unrestricted cubic splines. We then fitted a Cox proportional hazard regression model with unrestricted cubic splines for each exposure and a post estimation command to plot the hazard ratios (HRs) with 95% confidence intervals (CIs) for each unit of the exposure against the referent category.^22^ For linear relationships, we examined the associations between each adiposity measure, as a continuous covariate without further transformation, and CVD outcome with a Cox proportional hazard regression model. Prior to fitting the Cox regression models, we checked that the proportional hazards hold.

The start of follow up was 2 years post the date of the baseline visit for the associations with the incidence of CVD. Contributions to risk were censored at the date of the first outcome event of interest, death from any cause, or end of the follow-up period from those who remained alive and free of the outcomes of interest. We present fully adjusted models for age, socioeconomic status (Townsend quintiles and qualification), smoking, alcohol intake, MVPA, diabetes, and systolic blood pressure at baseline. We performed supplemental analysis without adjusting for diabetes and blood pressure because they can be mediators instead of confounders of the associations. We stratified the analysis by sex because of sex differences in body fat distribution and risk of CVD. We checked by fitting non-linear functions whether smoking or physical activity modified the associations between adiposity and CVD outcomes.

We carried out sensitivity analysis for the associations of adiposity measures with CVD events in healthy individuals by restricting the analyses to non-smokers and secondly non-smokers without comorbidities. The specific comorbidities considered were self-reported at baseline and included diabetes, cancer, chronic liver disorders, alcohol and substance abuse, eating disorders, depression, anxiety, inflammatory bowel disease, chronic obstructive pulmonary disease, and inflammatory disorders (e.g. rheumatoid arthritis, polyarthropathies). We additionally performed analysis without excluding the first 2 years of follow up. We also looked at the associations of adiposity measures with CVD mortality and morbidity separately and with a composite outcome including only ischaemic heart and cerebrovascular events (ICD I20-I25 and ICD I61-I67 and I69).

We reported the percentage of missing values of each variable. Participants with <2% missing values for a confounding variable or missing values on the exposures of interest were excluded from the analysis. None of the confounding variables had >2% missing values.

As secondary analysis, we compared the performance of each adiposity measure at predicting CVD events; we calculated the area under the curve of receiver operator characteristics (AUROCs) and the continuous net reclassification index (NRI) for each univariate model compared with that of BMI. We also looked how many participants were reclassified to lower (0–5% risk) or higher (5–10%) risk category depending on the adiposity measure used whether the outcome is present or absent.^23^ We also explored the correlations of each adiposity metric with each other by estimating all pairwise correlation coefficients.

All analyses were performed with Stata (version 14, StataCorp LP, College Station, TX, USA) and R (R version 3.3.1, https://www.r-project.org).

## Results

The study population comprised the 296 535 UK Biobank participants of white European descent without prevalent CVD at baseline who were followed up for an average of 5 years (interquartile range 4.3–5.6). Of this population 171 285 (57.8%) were women, 5667 (3.3%) of whom developed a CVD event, and 125 250 (42.2%) were men, 7187 (5.7%) of whom developed a CVD event, during the follow-up period. *Table **1* shows the cohort characteristics stratified by sex.

**Table 1:** Characteristics of the cohort stratified by sex

	Women without CVD at baseline	Men without CVD at baseline
*N*	171 285	125 250
Age (years)	55.2 ± 8.0	55.1 ± 8.2
BMI (kg m^−2^)	26.3 ± 4.7	27.1 ± 3.8
Missing values, *n* (%)	534 (0.3)	451 (0.4)
Waist circumference (cm)	81.0 (74.0–89.0)	94.0 (88.0–101.0)
Missing values, *n* (%)	393 (0.2)	338 (0.3)
Hip circumference (cm)	101.0 (96.0–107.0)	102.0 (98.0–106.0)
Missing values, *n* (%)	397 (0.2)	355 (0.3)
Waist-to-hip ratio	0.81 ± 0.07	0.92 ± 0.06
Missing values, *n* (%)	409 (0.2)	361 (0.3)
Waist-to-height ratio	0.51 ± 0.07	0.54 ± 0.06
Missing values, *n* (%)	457 (0.3)	406 (0.3)
Body fat mass (%)	36.0 (31.0–40.0)	24.0 (21.0–28.0)
Missing values, *n* (%)	2269 (1.3)	1737 (1.4)
Smoking, *n* (%)		
Current	15 562 (9.1)	15 693 (12.5)
Ex	53 210 (31.1)	43 047 (34.4)
Never	102 019 (59.6)	66 146 (52.8)
Missing values, *n* (%)	494 (0.3)	364 (0.3)
Alcohol intake, *n* (%)		
Daily	29 436 (17.2)	31 952 (25.5)
Three to four times a week	38 844 (22.7)	34 776 (27.7)
Once or twice a week	46 380 (27.1)	33 893 (27.1)
One to three times a month	22 545 (13.2)	11 282 (9.0)
Special occasions	22 030 (12.9)	7757 (6.2)
Never	11 960 (7.0)	5514 (4.4)
Missing values, *n* (%)	90 (0.1)	76 (0.1)
Qualifications, *n* (%)		
Higher degree	58 292 (34.0)	46 339 (37.0)
Any school degree	71 209 (41.6)	45 094 (36.0)
Vocational degrees	6911 (4.0)	10 577 (8.4)
Other qualifications	9052 (5.3)	4997 (4.0)
None of the above	24 524 (14.3)	17 316 (13.8)
Missing values, *n* (%)	1297 (0.8)	927 (0.7)
Townsend quintiles, *n* (%)		
1	36 710 (21.4)	27 367 (21.9)
2	36 059 (21.1)	26 392 (21.1)
3	35 566 (20.8)	25 579 (20.4)
4	34 102 (19.9)	24 308 (19.4)
5	28 649 (16.7)	21 448 (17.1)
Missing values, *n* (%)	199 (0.1)	156 (0.1)
MVPA (min day^−1^)	50 (10–90)	60 (20–110)
History of diabetes, *n* (%)	2605 (1.5)	3176 (2.5)
Missing values, *n* (%)	209 (0.1)	242 (0.2)
Systolic BP (mmHg)	130 (119–143)	137 (127–148)
Missing values, *n* (%)	270 (0.2)	146 (0.1)
Comorbidities, *n* (%)	34 818 (20.3)	18 592 (14.8)
CVD events (*n*)	5667	7187
IHD	1148	2599
Cerebrovascular	573	697

Data are presented as mean ± standard deviation or median (interquartile range) unless stated otherwise.

BMI, body mass index; CVD, cardiovascular disease; IHD, ischaemic heart disease; MVPA, moderate to vigorous physical activity.

The referent value for BMI was 22 kg m^−2^.^6^ Referent values that corresponded to BMI of 22 kg m^−2^ (by regressing the BMI on each adiposity measure) were for waist circumference 74 and 83 cm; for waist-to-hip ratio 0.78 and 0.88; for waist-to-height 0.38 and 0.42; for percentage body fat mass 30 and 18% for women and men, respectively.

**Figure 1:**
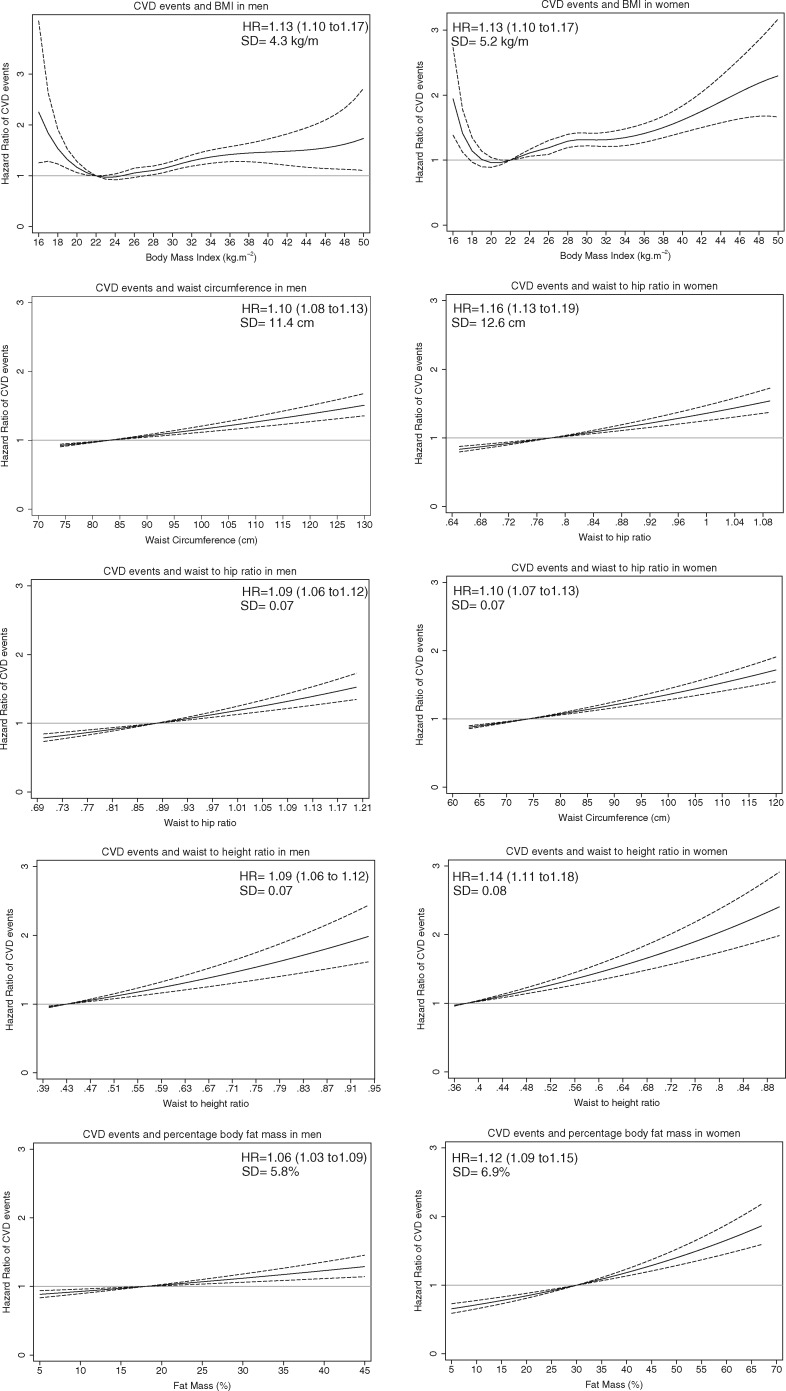
Hazard ratio of cardiovascular events in men and women in relation to body mass index, waist circumference, waist-to-hip ratio, waist-to-height ratio, and percentage body fat mass referent to the reference category. Hazard ratio (95% confidence intervals) per 1 SD increase in each adiposity marker are presented, for body mass index the hazard ratios correspond to those with a body mass index over 22 kg m^−2^. The grey line indicates hazard ratio of one at the referent category.

*Figure* *1* shows the adjusted HR and 95% CI for CVD events for each adiposity measure for women and men, respectively. Very low BMI (≤ 18.5 kg m^−2^) was associated with higher incidence of CVD and the lowest risk of CVD was exhibited at BMI of 22–23 kg m^−2^ and thereafter the incidence of CVD increased monotonically up to BMI of 35 kg m^−2^ for men and of 45 kg m^−2^ for women. Whereas, for the remaining adiposity measures the associations were log-linear where a higher adiposity was associated with greater risk of CVD events. Supplementary material online, *Figures S1* and *S2* presents the adjusted HR with 95% CI for non-smoker men and women, respectively, without comorbidities. For non-smoker men without comorbidities the higher risk at low BMI disappeared. Supplementary material online, *Figure S3* presents the adjusted HR with 95% CI for the participants without excluding those that their follow up was terminated within the first 2 years and the results are comparable with that in *Figure **1*. Supplementary material online, *Figure S4* presents the associations of adiposity measures with the composite outcome of ischaemic heart and cerebrovascular events and Supplementary material online, *Figures S5* and *S6* present the association of adiposity measures with fatal and non-fatal events separately, without the results changing substantially compared with that for the primary outcome of CVD events.

**Take home figure:**
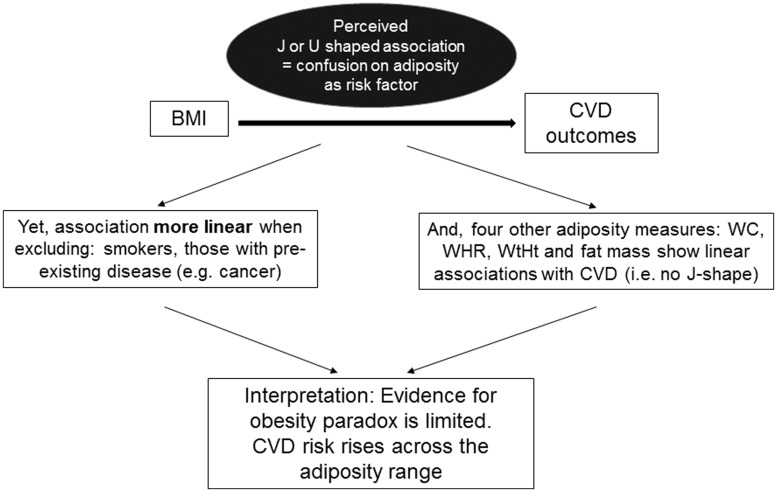
The obesity paradox is mainly due to the effect of confounding on BMI and disappears on other adiposity measures.

*Table* *2* presents the HR for CVD events per 1 SD increase in each adiposity measure in women and men without pre-existing disease at baseline. One SD increase in BMI (5.2 kg m^−2^ for women and 4.3 kg m^−2^ for men) was associated with a 13% higher (HR of 1.13, 95% CI 1.10–1.17) in both women and men in the risk of CVD events for BMI over 22 kg m^−2^. One SD increase in waist circumference (12.6 cm for women and 11.4 cm for men) was associated with a HR of 1.16 (95% CI 1.13–1.19) for women and 1.10 (95% CI 1.08–1.13) for men for CVD events. One SD increase in waist-to-hip ratio (0.07 for women and men) was associated with a 10% (HR of 1.10, 95% CI 1.07–1.13) and a 9% (HR of 1.09, 95% CI 1.06–1.12) increase in the risk of CVD events. One SD increase in waist-to-height ratio (0.08 for women and 0.07 from men) was associated with a 14% (HR of 1.14, 95% CI 1.11–1.18) and 9% (HR 1.06, 95% CI 1.06–1.12) increase in the risk of CVD events. Similarly, 1 SD increase in percentage body fat mass (6.9% for women and 5.8% for men) was associated with a HR of 1.12 (95% CI 1.09–1.15) in women and 1.06 (95% CI 1.03–1.09) in men. The associations became slightly stronger when we did not adjust for blood pressure and diabetes (Supplementary material online, *Table S1*).

**Table 2:** Adiposity markers and cardiovascular events (fatal and non-fatal) for individuals without pre-existing CVD at baseline

Exposures	Population (*n*)	Events (*n*)	Hazard ratio (95% CIs)	*P*-value
BMI				
<22 kg m^−2^				
Women	19 854	470	0.52 (0.34–0.78)	0.002
Men	5313	312	0.62 (0.40–0.96)	0.03
≥22 kg m^−2^				
Women	148 490	5048	1.13 (1.10–1.17)	<0.001
Men	117 735	6686	1.13 (1.10–1.17)	<0.001
Waist circumference				
Women	168 480	5529	1.16 (1.13–1.19)	<0.001
Men	123 155	7009	1.10 (1.08–1.13)	<0.001
Waist-to-hip ratio				
Women	168 465	5526	1.10 (1.07–1.13)	<0.001
Men	123 133	7009	1.09 (1.06–1.12)	<0.001
Waist-to-height ratio				
Women	168 420	5523	1.14 (1.11–1.18)	<0.001
Men	123 091	7004	1.09 (1.06–1.12)	<0.001
Percentage body fat				
Women	166 644	5459	1.12 (1.09–1.15)	<0.001
Men	121 791	6920	1.06 (1.03–1.09)	<0.001

The HRs correspond to 1 SD increase in each adiposity marker. HR are fully adjusted for age, diabetes, systolic blood pressure, moderate to vigorous physical activity, Townsend quintile, qualifications, alcohol intake, and smoking. Analyses are stratified by sex. HRs for BMI are shown for <22 kg m^−2^ and ≥22 kg m^−2^ because of the *U*-shape relationship between BMI and incidence of CVD.

Supplementary material online, *Table S2* presents the AUROC and NRI for each adiposity measure used as predictor of CVD event compared with the model using BMI. Waist circumference, waist-to-hip-ratio, and waist-to-height ratio present marginally better discriminatory characteristics than BMI in predicting a CVD outcome, whereas body fat mass have limited value compared with BMI. Supplementary material online, *Table S3* shows the correlation coefficients for each pair of adiposity measures; BMI correlates moderately with other measures (e.g. correlation coefficient of 0.43 with waist-to-hip ratio to 0.87 with waist-to-height ratio).

Moderate to vigorous physical activity did not modify the associations between adiposity measures and CVD outcomes (*P* for interaction >0.05 for each association) so we do not present stratified analyses per physical activity level. Smoking was an effect modifier for the association between BMI and CVD in men without CVD at baseline (*P* = 0.001).

## Discussion

We demonstrated that the J-shape association of BMI with the incidence of CVD, which is in accordance with the findings from other cohorts,^6^ almost disappeared in subgroup analysis in participants without comorbidities or in the non-smokers, whereas the associations of the remaining adiposity measures with the incidence of CVD were generally unchanged before or after such adjustments. These observations collectively suggest that the observed detrimental ‘impact’ of low BMI on CVD outcomes is likely a result of confounding.^24^ Our study has extended previous work, which looked only at associations of BMI with health outcomes by demonstrating novel associations with other well validated measures of adiposity and fat distribution. The size and wealth of information of the UK Biobank enabled comprehensive subgroup analyses and evidence of attenuation of the impact of confounding on the previously reported association of BMI with CVD. Our findings strongly reaffirm that being overweight heightens the risk of CVD and that individual should seek to keep their weights as close to recommended levels to lessen their risks of CVD. These findings may therefore have implications for future guidelines.

The impact of confounding with smoking, on the associations of BMI with CVD has been demonstrated previously in smaller cohort studies.^25^^,^^26^ Weight loss prior to diagnosis of disease is well reported.^15^ In addition, individuals with comorbidities (e.g. rheumatoid arthritis) are more likely to have decreased body mass as a result of lean mass loss^27^ but they have greater risk of CVD.^28^ Therefore, the disproportionally greater number of ill people in the low categories of BMI that are at increased risk of CVD events inflate the HR of CVD events in this BMI category. We extended our analysis to the associations of other adiposity measures, that are less likely to be affected by pre-existing illness (since illness is associated with lean rather than fat mass loss^27^), and we demonstrated a linear association between central and total body fat with the risk of CVD. That a study using mendelian randomisation analysis^29^ did not replicate the association of low BMI with greater risk of CVD supports our assertion that the association of low BMI with greater risk of CVD is not causal. On the contrary, mendelian randomization analysis supported the causal relationship of higher abdominal adiposity (measured by waist-to-hip ratio) with greater risk of coronary heart disease.^30^

Previous evidence has suggested that measures of abdominal adiposity have stronger associations, than BMI, with the incidence of primary myocardial infarction^31^ or CVD mortality,^32^ which may be mediated through the impact of visceral fat (or other ectopic fats) on adverse metabolic profile.^33^ Our findings show that increasing abdominal adiposity is associated with a higher hazard for CVD, that the magnitude of the associations are comparable with that, or potentially slightly superior to BMI in predicting future CVD, however, one accepts we did not have lipid values to adjust for which may capture some of this excess predictive ability. It is possible that measures of central adiposity may complement or be useful alternatives to the use of BMI in CVD risk stratification, especially for those individuals with low BMI. We acknowledge that BMI is a more easily reproducible than central adiposity measures.^4^

Our study importantly extends previous analyses of studies looking at the relationship between adiposity and CVD.^4^^,^^5^ It is the largest prospective study linking increased body fat measured with bioimpedance with future CVD events and refutes previous conflicting findings, which were either based on smaller cohorts including participants with existing disease^34^^,^^35^ or where the effect of body fat was diluted by over-adjustment for other adiposity markers.^35^^,^^36^ Our findings in respect to BMI and waist circumference are in line with accumulated data from prospective studies, not inclusive of the UK Biobank,^4–6^ adding external validity that their association with CVD risk are genuine. Our relatively modest effect size of the associations compared with that in the aforementioned meta-analyses might be attributed to our additional adjustment for physical activity which has attenuated the associations, the relatively shorter follow-up duration and the contemporary nature of the cohort which is likely to be associated with better CVD prevention.

### Strengths and weaknesses

UK Biobank is a unique resource, which includes a large contemporary cohort with consistent measures of adiposity and homogenously defined outcomes and risk factors. The ongoing linkage with death and hospital registries minimises the number of cases that are lost from follow up. It is the largest study linking body fat measured with a validated technique with the incidence of CVD. We used robust analysis examining non-linearity and treating exposures as a continuum rather than categorising them at arbitrary levels. Our landmark and sensitivity analyses contribute to minimise effect of bias of undiagnosed disease, comorbidities and smoking on adiposity, however, we accept there may still be residual confounding. We restricted our analysis to participants of White European disease as ethnicity modifies the association between adiposity and vascular health^5^ and there was not adequate number of events for Subgroup analyses of other ethnic groups.

We acknowledge that our study has some limitations; it can be argued that the low response rate (5.5%) to the UK Biobank recruitment may have introduced a healthy responder bias to the analyses. While this would limit the ability to generalize prevalence rates which is beyond the scope of the study, estimates of the magnitude of associations regarding disease or mortality risk in the current study are not expected to be affected by this.^37^^,^^38^ That the shape of associations for BMI and waist circumference in our study are comparable to that in previous reports^4–6^ provides additional assurance that the low response rate has not had a substantial impact on the findings. We did not have access to lipid or glycaemia biomarkers; however, they are likely to be additional mediators of the association between adiposity and CVD, not confounders.^39^ Measurement of fat with impedance is a well-validated technique,^40^ and further analyses should be undertaken using imaging data (still in its infancy in UK Biobank) on abdominal fat storage and ectopic fat depots.

## Conclusion

In conclusion, increasing adiposity, whether total body and ‘central’ adiposity measures, have generally adverse associations with CVD outcomes in middle-aged men and women. Public health campaigns should emphasize the importance of an individual intentionally maintaining as lean a phenotype as possible to gain maximum CVD benefits. The association of BMI with CVD is more susceptible to bias rather than other adiposity measures and, therefore, health care professionals should challenge any public misconception of some ‘protective’ effect of fat on CVD risk.

## Supplementary material

Supplementary material is available at *European Heart Journal* online.

## Supplementary Material

Supplementary Data
