# Supplementary material for: The impact of confounding on the associations of different adiposity measures with the incidence of cardiovascular disease: a cohort study of 296 535 adults of white European descent
Source: Eur Heart J. 2018 Mar 16;39(17):1514–20. doi: 10.1093/eurheartj/ehy057 (PMC5930252; doi:10.1093/eurheartj/ehy057)
Supplement: Supplementary Data [file ehy057_suppl_data.zip › Revised_Supplemental_Table 2.docx]

Supplemental Table 2. Discrimination characteristics of each adiposity measure for prediction of cardiovascular events. The continuous net reclassification index (NRI) has been estimated for each univariate model referent to the model for body mass index (BMI). In addition, reclassification tables for each variable are presented (cut off points of risk 5% and 10%).

Abbreviations: AUROC: area under the curve of the receiver operator characteristics curve

| **Predictor** | **AUROC (95% CI)** | **NRI continuous (95% CI)** |
| --- | --- | --- |
| **BMI** | 0.553 (0.548 to 0.558) | Reference |
| **Waist Circumference** | 0.587 (0.582 to 0.592) | 0.27 (0.25 to 0.29) |
| **Waist to hip ratio** | 0.595 (0.590 to 0.600) | 0.25 (0.24 to 0.27) |
| **Waist to height ratio** | 0.576 (0.571 to 0.581) | 0.23 (0.21 to 0.25) |
| **Body fat mass** | 0.522 (0.517 to 0.527) | -0.10 (-0.12 to -0.08) |

**Reclassification Table for Waist Circumference**

Outcome: absent

|  | **Model with Waist circumference** | |  |
| --- | --- | --- | --- |
| **Model with BMI** | **0 to 5% risk** | **5 to 10% risk** | **% reclassified** |
| **0 to 5% risk** | 202,567 | 44,582 | 18 |
| **5 to 10% risk** | 5,708 | 26,118 | 18 |

Outcome: present

|  | **Model with Waist circumference** | |  |
| --- | --- | --- | --- |
| **Model with BMI** | **0 to 5% risk** | **5 to 10% risk** | **% reclassified** |
| **0 to 5% risk** | 7,869 | 2,895 | 27 |
| **5 to 10% risk** | 190 | 1,634 | 10 |

**Reclassification Table for Waist to hip ratio**

Outcome: absent

|  | **Model with Waist to hip ratio** | |  |
| --- | --- | --- | --- |
| **Model with BMI** | **0 to 5% risk** | **5 to 10% risk** | **% reclassified** |
| **0 to 5% risk** | 180,439 | 66,567 | 27 |
| **5 to 10% risk** | 15,993 | 16,179 | 50 |

Outcome: present

|  | **Model with Waist to hip ratio** | |  |
| --- | --- | --- | --- |
| **Model with BMI** | **0 to 5% risk** | **5 to 10% risk** | **% reclassified** |
| **0 to 5% risk** | 6,533 | 4,219 | 39 |
| **5 to 10% risk** | 688 | 1,151 | 37 |

**Reclassification Table for Waist to height ratio**

Outcome: absent

|  | **Model with Waist to height ratio** | |  |
| --- | --- | --- | --- |
| **Model with BMI** | **0 to 5% risk** | **5 to 10% risk** | **% reclassified** |
| **0 to 5% risk** | 221,667 | 25,482 | 10 |
| **5 to 10% risk** | 4,607 | 27,579 | 14 |

Outcome: present

|  | **Model with Waist to height ratio** | |  |
| --- | --- | --- | --- |
| **Model with BMI** | **0 to 5% risk** | **5 to 10% risk** | **% reclassified** |
| **0 to 5% risk** | 9,086 | 1,678 | 16 |
| **5 to 10% risk** | 189 | 1,662 | 10 |
