# Supplementary material for: The impact of confounding on the associations of different adiposity measures with the incidence of cardiovascular disease: a cohort study of 296 535 adults of white European descent
Source: Eur Heart J. 2018 Mar 16;39(17):1514–20. doi: 10.1093/eurheartj/ehy057 (PMC5930252; doi:10.1093/eurheartj/ehy057)
Supplement: Supplementary Data [file ehy057_suppl_data.zip › Supplemental_Table 1.docx]

| **Exposures** | **Population (n)** | **Events (n)** | **Hazard ratio (95% CIs)** | **P-value** |
| --- | --- | --- | --- | --- |
| **BMI**  **< 22 kg.m^-2^**  Women  Men  ≥ **22 kg.m^-2^**  Women  Men | 19,881  5,329  148,815  117,975 | 472  313  5,061  6,711 | 0.54 (0.36 to 0.81)  0.65 (0.42 to 1.01)  1.15 (1.12 to 1.19)  1.16 (1.13 to 1.19) | 0.003  0.06  <0.001  <0.001 |
| **Waist circumference**  Women  Men | 168,834  123,414 | 5,545  7,036 | 1.18 (1.15 to 1.21)  1.13 (1.10 to 1.17) | <0.001  <0.001 |
| **Waist to hip ratio**  Women  Men | 168,819  123,391 | 5,542  7,035 | 1.12 (1.09 to 1.14)  1.11 (1.08 to 1.14) | <0.001  <0.001 |
| **Waist to height ratio**  Women  Men | 168,772  123,348 | 5,538  7,030 | 1.17 (1.13 to 1.20)  1.12 (1.09 to 1.15) | <0.001  <0.001 |
| **% Body fat mass**  Women  Men | 166,986  122,036 | 5,474  6,945 | 1.14 (1.10 to 1.17)  1.08 (1.06 to 1.11) | <0.001  <0.001 |

Supplemental Table 1: Adiposity markers and cardiovascular events (fatal and non-fatal) for individuals without pre-existing CVD at baseline. The HRs correspond to one SD increase in each adiposity marker. HR are fully adjusted for age, moderate to vigorous physical activity, Townsend quintile, qualifications, alcohol intake and smoking. Analyses are stratified by sex. HRs for BMI are shown for < and ≥ 22 kg.m^-2^ because of the U-shape relationship between BMI and incidence of CVD.
