# Supplementary material for: The impact of confounding on the associations of different adiposity measures with the incidence of cardiovascular disease: a cohort study of 296 535 adults of white European descent
Source: Eur Heart J. 2018 Mar 16;39(17):1514–20. doi: 10.1093/eurheartj/ehy057 (PMC5930252; doi:10.1093/eurheartj/ehy057)
Supplement: Supplementary Data [file ehy057_suppl_data.zip › Supplemental_Table 3.docx]

|  | **BMI** | **Waist circumference** | **Waist to hip ratio** | **Waist to height ratio** | **% body fat mass** |
| --- | --- | --- | --- | --- | --- |
| **BMI** | 1 |  |  |  |  |
| **Waist circumference** | 0.79 | 1 |  |  |  |
| **Waist to hip ratio** | 0.43 | 0.82 | 1 |  |  |
| **Waist to height ratio** | 0.87 | 0.92 | 0.71 | 1 |  |
| **% body fat mass** | 0.55 | 0.19 | -0.16 | 0.42 | 1 |

**Supplemental Table 3:** Pairwise correlation coefficients between different adiposity measures.
