# Supplementary figures and images for: The impact of confounding on the associations of different adiposity measures with the incidence of cardiovascular disease: a cohort study of 296 535 adults of white European descent
Source: Eur Heart J. 2018 Mar 16;39(17):1514–20. doi: 10.1093/eurheartj/ehy057 (PMC5930252; doi:10.1093/eurheartj/ehy057)

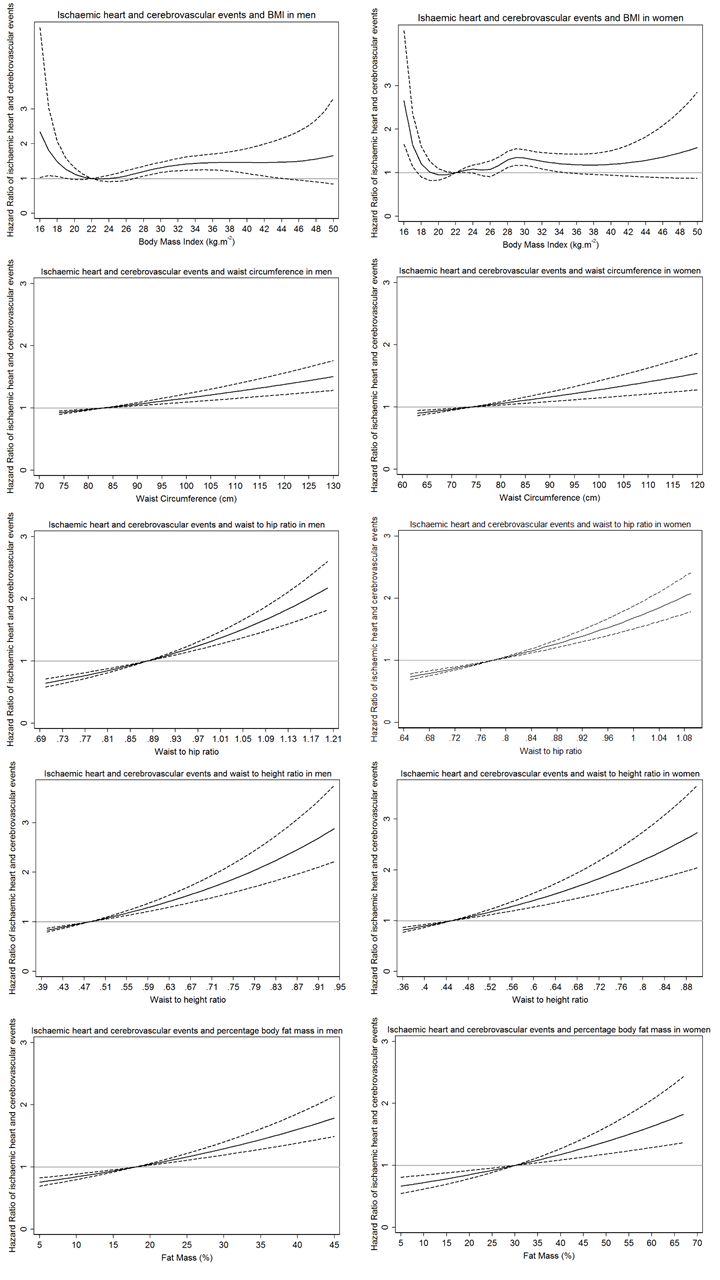

Supplement: Supplementary Data [file ehy057_suppl_data.zip › Supplemental_4_composite.tif]

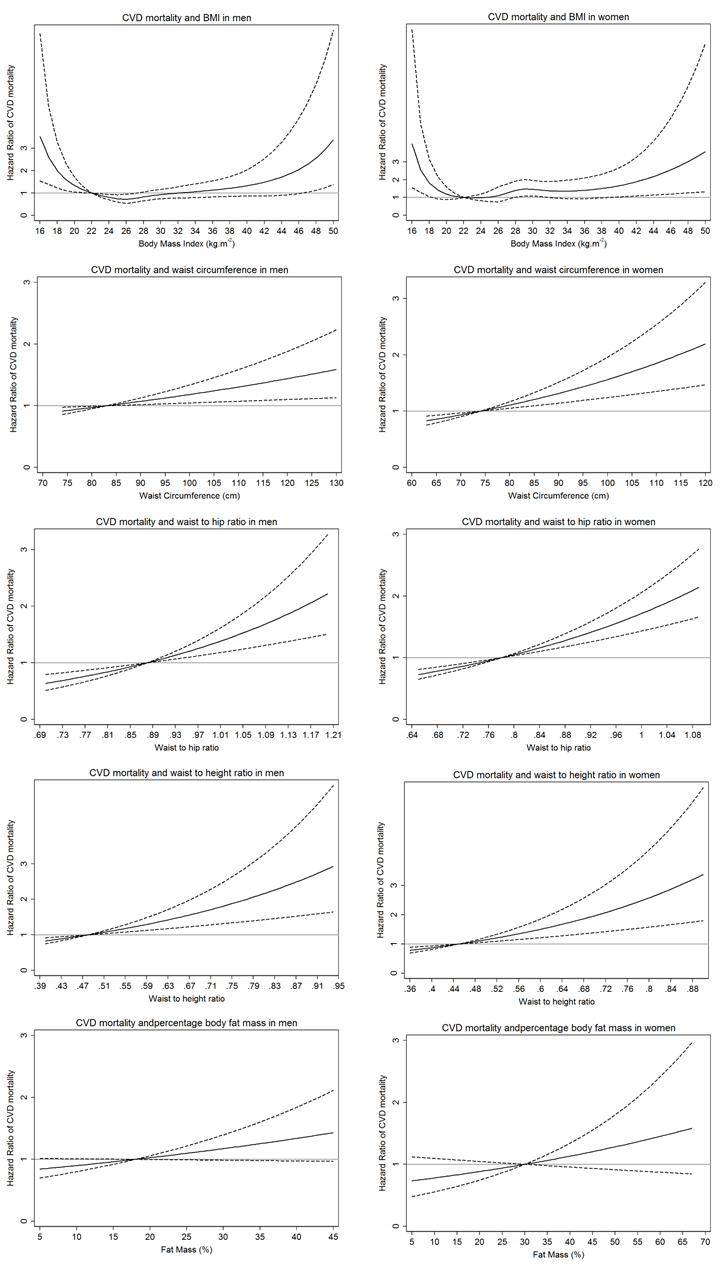

Supplement: Supplementary Data [file ehy057_suppl_data.zip › Supplemental_5_mortality.tif]

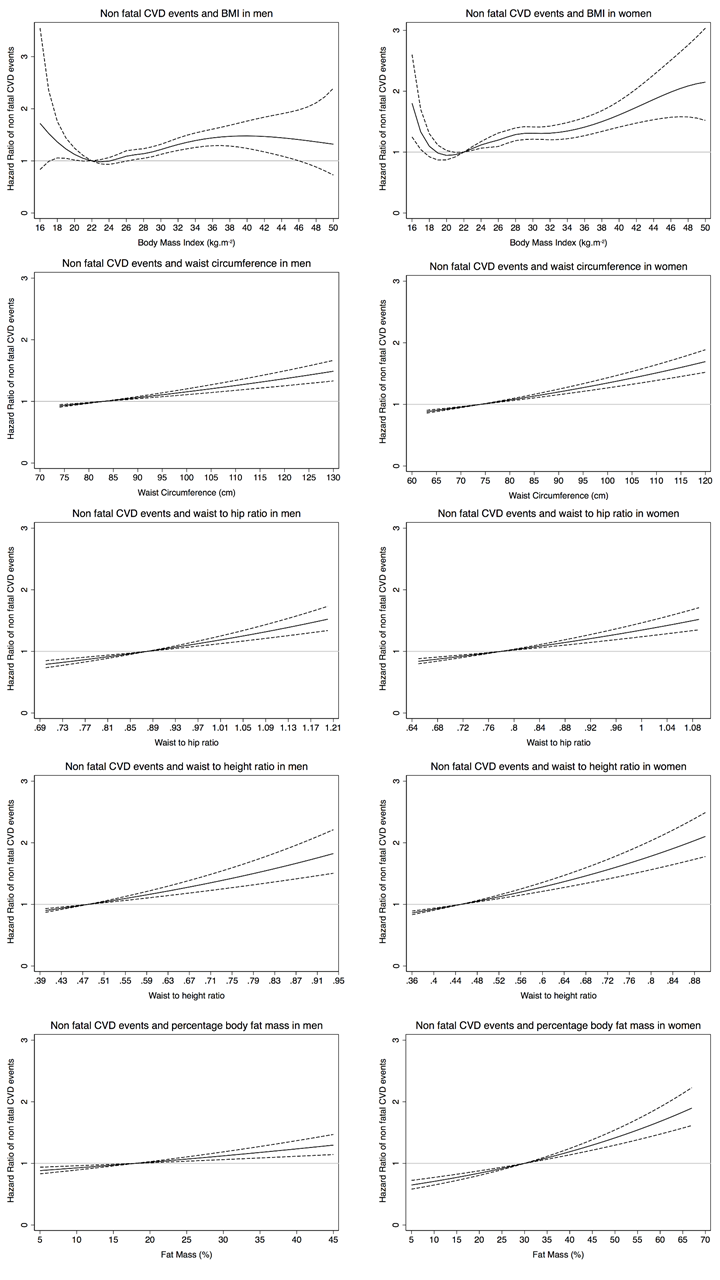

Supplement: Supplementary Data [file ehy057_suppl_data.zip › Supplemental_6_morbidity.tif]

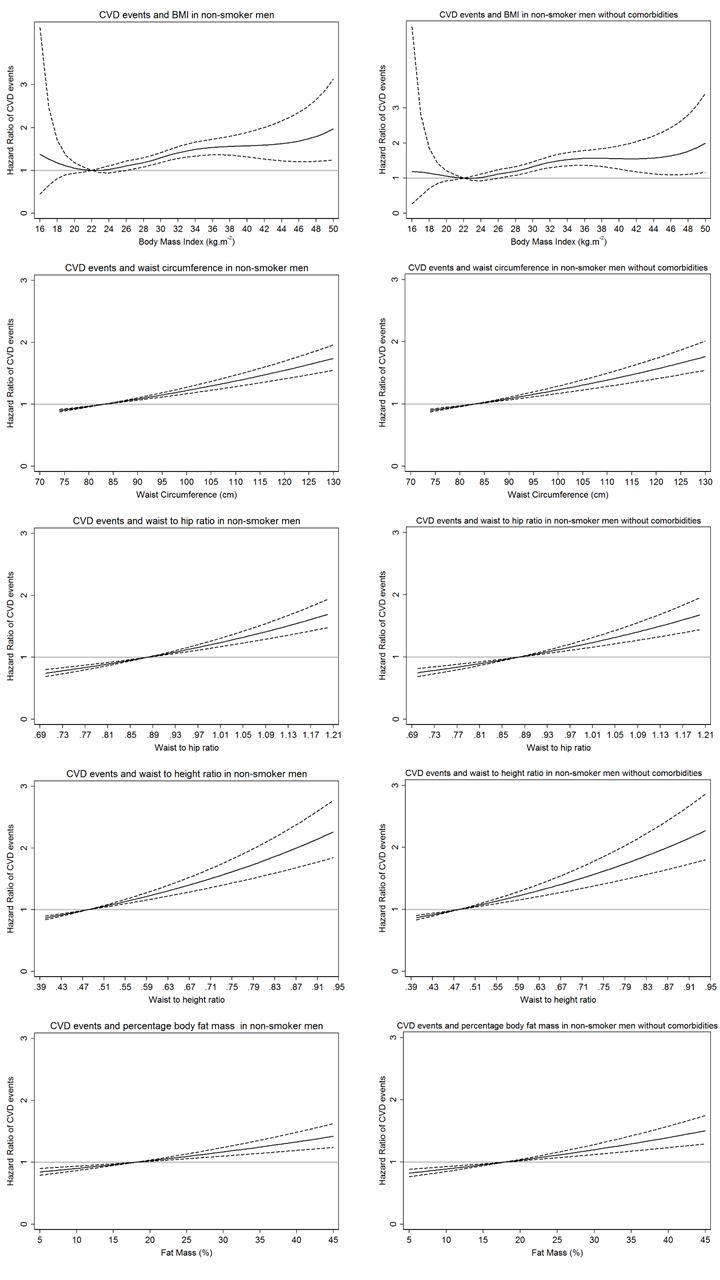

Supplement: Supplementary Data [file ehy057_suppl_data.zip › Supplemental_Fig1.tif]

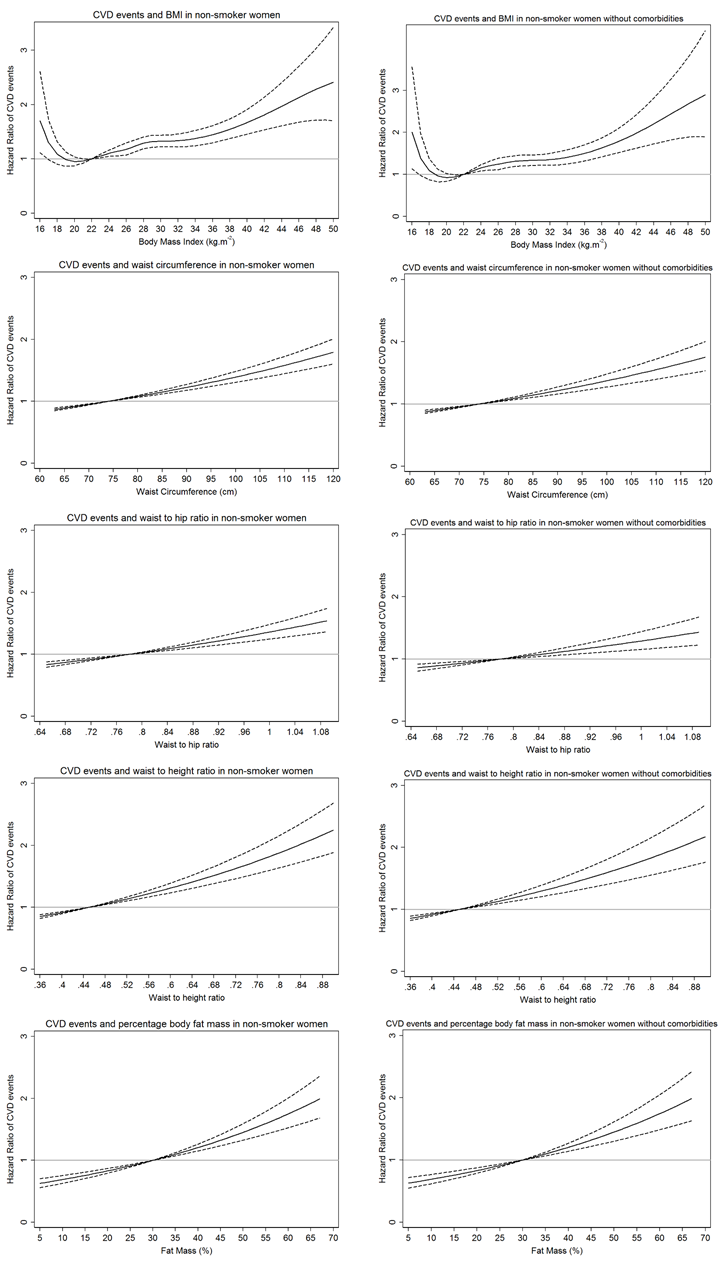

Supplement: Supplementary Data [file ehy057_suppl_data.zip › Supplemental_Fig2.tif]

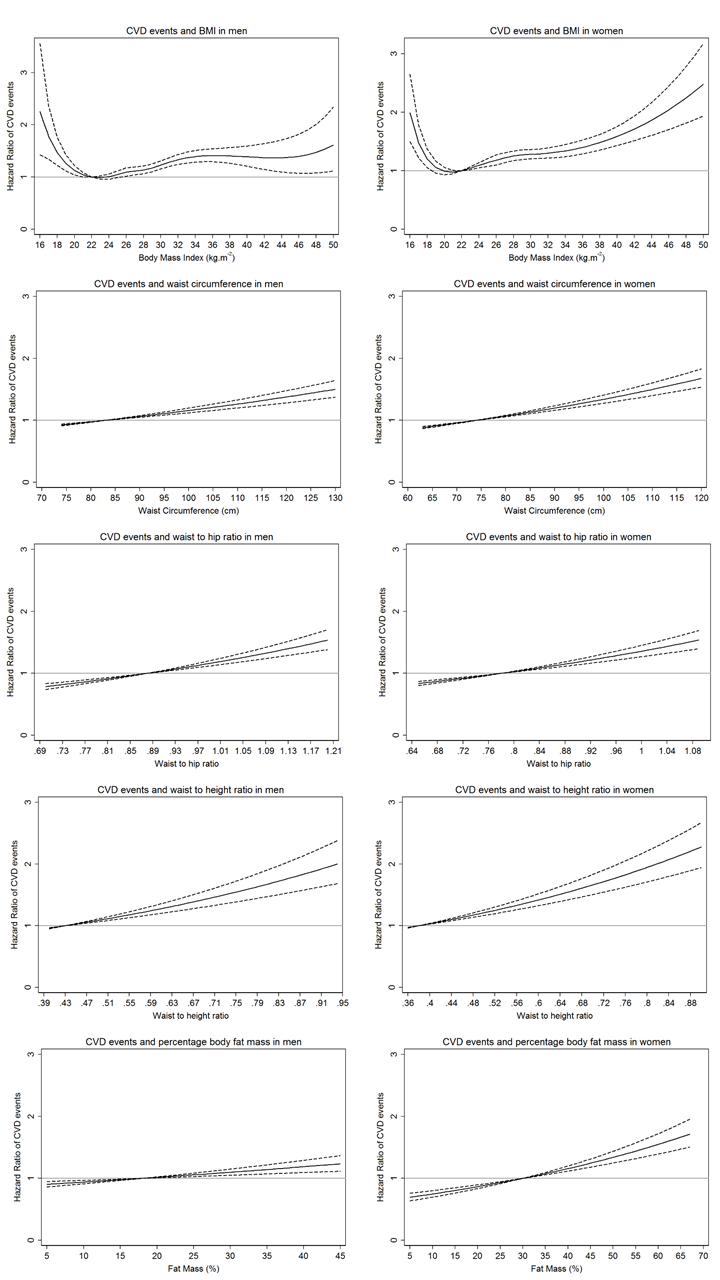

Supplement: Supplementary Data [file ehy057_suppl_data.zip › Supplemental_Fig3.tif]
